# Skeletal stem cells and their contribution to skeletal fragility: senescence and rejuvenation

**DOI:** 10.1007/s10522-015-9623-7

**Published:** 2015-10-28

**Authors:** Abdullah Aldahmash

**Affiliations:** Stem Cell Unit, Department of Anatomy, College of Medicine, King Saud University, Riyadh, Saudi Arabia; Department of Endocrinology and Metabolism, University Hospital of Odense, 5000 Odense, Denmark

**Keywords:** Skeletal stem cells, Mesenchymal stem cells, Cellular senescence, Cell therapy, Osteoporosis

## Abstract

Age-related osteoporotic fractures are major health care problem worldwide and are the result of impaired bone formation, decreased bone mass and bone fragility. Bone formation is accomplished by skeletal stem cells (SSC) that are recruited to bone surfaces from bone marrow microenvironment. This review discusses targeting SSC to enhance bone formation and to abolish age-related bone fragility in the context of using stem cells for treatment of age-related disorders. Recent studies are presented that have demonstrated that SSC exhibit impaired functions during aging due to intrinsic senescence-related changes as well as the presence of senescent microenvironment. Also, a number of approaches aiming at increasing bone formation through targeting SSC and that include systemic SSC transplantation, systemic SSC targeting using aptamers or antibodies, use of therapeutic screteome and tissue engineering approaches will be presented and discussed.

## Introduction

Aging is the most important risk factor for fragility fracture leading to the highly prevalent disease osteoporosis. Osteoporosis is defined as a disease of low bone mass and bone architectural deterioration that lead to bone fragility (please see review (Drake et al. 2015). Bone fragility is caused by a multitude of factors including sex hormone deficiency, insufficient nutritional intake of calcium and vitamin D, immobilization as well as multiple biological changes occurring in the aging human organism that have been reviewed in (Kassem and Marie 2011; Marie and Kassem 2011). All these factors mediate their influence on bone by inducing changes in bone remodeling mechanisms.

Bone remodeling is a cyclic regenerative process taking place in adult human skeleton, that aims at removing “old bone” filled with fatigue micro-fractures, by bone resorbing osteoclastic cells and replacing it with young bone of better biomechanical properties through the action of bone forming osteoblastic cells (Parfitt et al. 2011). Bone remodeling leads to full regeneration of the whole skeleton every 10 years during the adult human life (Manolagas and Parfitt 2010). Bone remodeling rate increases during sex steroid deficiency states, aging and in some osteoporotic patients (Manolagas and Parfitt 2010). Several histomorphometric studies, that examined the dynamics of bone formation and bone resorption in aged patients with osteoporosis revealed the presence of bone formation defect caused by poor recruitment of osteoblastic cells or defective osteoblastic functions as the main pathophysiological mechanism (Parfitt et al. 2011).

## Skeletal stem cells (SSC) definition and functions

A number of recent studies have re-confirmed the general concept that bone formation during bone remodeling is accomplished by recruitment of skeletal stem cells (SSC) to bone formation surfaces. Genetic studies of SSC and osteoblast ablation in mice demonstrated a significant decrease in bone formation (Worthley et al. 2015). Histological studies of adult human bone show that SSC are recruited from a “canopy”/perivascular cells/pericytes located near the bone formation sites (Kristensen et al. 2014) which coincides with the assumed in vivo location of SSC (Crisan et al. 2008). While the name SSC is commonly used to describe bone marrow stem cells with bone forming capacity, the same cell population has been termed in the literature by a variety of other names e.g. bone marrow mesenchymal stem cells (MSC) or bone marrow stromal stem cells. In this review, we will keep the name SSC as suggested by a recent review (Kassem and Bianco 2015).

In vitro, SSC are cultured from bone marrow aspirates and enriched in through selective adherence to plastic surfaces (Rickard et al. 1996). Several studies have reported the possibility of using single or a combination of cell surface markers to isolate SSC prospectively from bone marrow aspirates e.g. CD146, CD271, Stro-1 or in mice: Sca-1, Gremlin 1, alpha V integrin (Chan et al. 2015; Gronthos et al. 1994; Simmons and Torok-Storb 1991; Tormin et al. 2011; Worthley et al. 2015). SSC can differentiate in vitro using standard assays that manipulate the cellular microenvironment, into osteoblasts, adipocytes and chondrocytes (termed tri-lineage differentiation) (Abdallah et al. 2005; Rickard et al. 1996). While some studies have reported the ability of SSC to differentiate into non-mesodermal cells e.g. hepatocytes or neuronal cells, these studies are controversial and not verified in vivo. Evidence for “stemness” of SSC is however is based on demonstrating the ability of SSC when implanted subcutaneously in immune deficient mice to form bone and bone marrow organ (Abdallah et al. 2008) and to maintain this ability during serial transplantation studies (Li et al. 2014; Sacchetti et al. 2007).

SSC-like cells have been isolated from a variety of tissues including muscle, adipose tissue, skin and umbilical cord blood (Al-Nbaheen et al. 2013; Rosada et al. 2003). These SSC-like cells exhibit variable efficiencies for tri-lineage differentiation in vitro, however they are poor at forming bone and bone marrow organ when transplanted in vivo and their molecular signatures based on global gene expression profiling differ significantly from bone marrow SSC (Al-Nbaheen et al. 2013). Thus, the bone fide bone forming SSC is the bone marrow derived population.

### Are SSC present in the circulation?

Some groups have demonstrated the presence of SSC in peripheral blood (Kuznetsov et al. 2001) and umbilical cord blood (Rosada et al. 2003), although at very low number compared to their presence in bone marrow and these cells exhibit a limited ability for in vivo bone formation (for review please see Pignolo and Kassem 2011). In parabiosis experiments, the contribution of a circulating population of osteoblastic cells or SSC to bone formation during fracture healing has been variable and generally few osteoblastic cells were identified within the fracture callus as derived from the circulation (Boban et al. 2010).

### Non-progenitor functions of SSC

In addition to their ability for multi-lineage differentiation, an additional aspect of SSC biology that is relevant to tissue regeneration and bone formation within the context of aging organism is their ability to secrete large number of regeneration enhancing molecules as reviewed in (Caplan and Correa 2011). This notion is based on the observed positive therapeutic effects on tissue regenerations observed in clinical trials employing bone marrow SSC or SSC-like cells e.g. trials for cardiac regeneration, cartilage regeneration and for treatment of graft-versus-host disease (Gvh). The effects observed in these conditions, can’t be explained by differentiation to resident cells since the number of SSC integrated in the tissues is very small. Using proteomic studies, a number of studies have dissected the secreted factors produced by SSC and reported the presence of a large number of growth factors, inflammation modulatory factors. Our group has recently reported a quantitative proteome profile of secreted factors by SSC at the undifferentiated state and during differentiation into osteoblastic cells. Examining the list of the secreted factors suggest a complex and multifaceted functions of SSC (Kristensen et al. 2012). One of the current areas of active research within the SSC field is to determine the functional and biological relevance of these secreted factors in relation to SSC role in tissue regeneration and bone formation.

## Age-related changes in SSC

Several major theories have been put forward in the field of biogerontology to explain the pathophysiology of aging processes (Rattan 2006, 2012). In the skeletal biology and SSC biology fields, some of these theories have been tested (Table 1) [also see review (Fukada et al. 2014)]. Generally, two approaches have been employed in these studies. The first is to isolate and establish in vitro SSC cultures from young and old donors and to study the effect of donor age on SSC number, response to differentiation signals and the presence of intrinsic intracellular signaling defects as well as the presence of donor age-associated changes. The original studies have been reviewed in (Kassem and Marie 2011). These experiments have been performed on cells isolated from mice, rats and humans. The second types of studies are based on analysis of SSC isolated from genetic mice models of aging and accelerated aging (please see below). The main results of these two types of studies will be discussed here.

**Table 1:** Examples of studies on skeletal stem cell aging and corresponding specific theories of aging

Theory of aging	Target mechanism(s)	References
Free radical damage	Oxidative stress and cell damage	Manolagas (2010), Manolagas and Almeida (2007), Nojiri et al. (2011)
Telomere shortening	Telomeric DNA damage and associated events	Saeed et al. (2011, 2015), Simonsen et al. (2002)
Somatic mutation	DNA repair	Barnhoorn et al. (2014), Chen et al. (2013)
Endocrine control	Endocrine homeostatic mechanisms	Abdallah et al. (2006), Baht et al. (2015), Conboy et al. (2005), Loffredo et al. (2013)

The in vitro studies of donor-age effects on SSC have reported highly variable results. The discrepancies can be attributed to differences in donor characteristics, site of obtaining bone marrow aspirates; methods of establishing SSC cultures and the absence of standardized criteria for defining SSC in vitro (Bellantuono et al. 2009). However, careful review of the reported studies reveals the following consistent findings. First, in humans the number SSC decreases between childhood/adolescence and adulthood and that the number of SSC is stable from 30 years of age and afterwards (Choumerianou et al. 2010; Stenderup et al. 2001). Second, exposing SSC obtained form elderly donors to “stress conditions” reveals molecular defects that are undetectable at steady state conditions. For example, cells obtained from elderly persons exhibit a decrease in vitro life span (so called Hayflick limit) when compared with cells obtained from young donors (Stenderup et al. 2003) and increased susceptibility to oxidative stress (Kasper et al. 2009) as well as impaired response to mitogenic/differentiation signals [reviewed in (Bellantuono et al. 2009; Kassem and Marie 2011)]. One caveat is that stem cells exist in vivo in quiescent “protected” state and may thus be protected from excessive proliferation (Rumman et al. 2015).

Studies of SSC from genetically induced accelerated aging in mice have also provided insight into the specific molecular defects contributing to age-related impairment of SSC functions. A number of in vivo mice models have been developed to study the contribution of a specific gene or a signaling pathway on the aging phenotype and some of these studies have reported evidence for increased bone fragility and osteoporosis (Marie 2014). Some examples will be presented here. Telomerase deficient mice with very short telomeres exhibit decreased bone mass and osteoporotic phenotype caused by deficiency in SSC number and impaired SSC differentiation into osteoblasts (Saeed et al. 2011). Telomere shortening has been proposed as a central mechanism mediating cellular senescence and consequently organismic aging [for review please see (Blasco 2007)]. Werner syndrome is a premature aging diseases caused by mutation in WRN gene needed for efficient DNA repair mechanisms. WRN deficient mice exhibit accelerated aging phenotype including osteoporosis and impaired differentiation of SSC (Pignolo et al. 2008). Mice deficient in cytoplasmic copper/zinc superoxide dismutase gene [CuZn-SOD, encoded by the Sod1 gene; Sod1(−/−)] that leads to increased production of reactive oxygen species (ROS), exhibit osteoporotic phenotype with increased bone fragility and impaired osteoblastic cell functions (Nojiri et al. 2011). Age-related accumulation of DNA and macromolecular damage cause by oxidative stress and reactive oxygen species, has been reported to play an important role in bone aging (Manolagas and Almeida 2007; Manolagas and Parfitt 2013). Genetically modified mice with DNA repair defects exhibit skeletal fragility and osteoporotic phenotype (Barnhoorn et al. 2014; Chen et al. 2013). These studies suggest that we need to approach SSC senescence and skeletal fragility as part of the generalized aging phenotype of the whole organism (Kassem and Marie 2011). They also provide framework for novel approach for prevention and treatment of senescent SSC.

## Age-related changes in SSC microenvironment

Cellular homeostatic mechanisms depend on hormone signaling and it is plausible that changes in hormonal “microenvironment” has long term consequences on stem cell aging and SSC aging. A number of studies have provided support for this hypothesis. Sera obtained from elderly donors exert inhibitory effects on osteoblast differentiation of SSC (Abdallah et al. 2006) and biological functions of a wide variety of cell types (Kondo et al. 1988). Aging is associated with a multitudes of changes in the neuroendocrine system including significant changes in pituitary hormones and sex steroids and thus endocrine replacement therapy has been a very popular form of anti-aging therapy with aim of restoring hormone levels to young range. Hormones used in anti-aging therapies include growth hormone, insulin-like growth factor 1 (IGF-1), sex steroid, dehydroepiandrosterone (DHEA) with unfortunately limited anti-aging effects (Bao et al. 2014). A number of recent studies have provided a strong credence to “endocrine theory of aging”. The most impressive is evidence from multiple laboratories employing heterochronic parabiosis which is an experimental procedure that creates surgically a connection between the blood circulations of animals of different ages. Employing this technique, a number of investigators reported reversal of several of the age-related pathologies of the aged mice when “parabiosed” with young mice including decreased cardiac hypertrophy, increased muscle regeneration capacity, increased neurogenesis and neural cell functions, increased beta cell replication and improved fracture healing (Baht et al. 2015; Conboy et al. 2005; Loffredo et al. 2013). These experiments suggest that tissue-levels defects observed during aging and more pronounced in age-related diseases are caused by the presence of “aging-inducing factors” or absence of “pro-youthful factors”. Growth differentiation factor 11 (GDF11) has been suggested to play a role, as a youthful factor (Loffredo et al. 2013). GDF11 is a member of the transforming growth factor β superfamily. It has been reported that serum levels of GDF11 decrease with age. However, injections of recombinant GDF11 (rGDF11) into old mice caused partial rejuvenation suggesting the presence of additional circulating factors (Loffredo et al. 2013). Further support of the endocrine theory of aging is derived from two therapeutic interventions that have been shown to decrease the rate of aging and extend life span in experimental animals: calorie restriction and rapamycin treatment. Both converge on nutrition-associated hormone signaling pathways including insulin and insulin-IGF-1 signaling (Oh et al. 2014). Also, recently, alpha Klotho (Klotho) gene and protein coding for a circulating protein first identified as factor associated with premature aging and with a role in calcium homeostasis (Imura et al. 2007). Klotho deficient mice exhibit a reduced lifespan and accelerated aging phenotype including bone fragility and osteoporosis (Kuro-o et al. 1997). Mice overexpressing the Klotho gene exhibit extended lifespan (Kurosu et al. 2005) and ablation of p16 (INK4a) reverses the accelerated aging phenotype in mutant mice homozygous for a hypomorphic allele of the α-klotho gene through restoration of the expression of Klotho gene (Sato et al. 2015). Interestingly, the soluble Klotho protein interacts with multiple hormonal signaling pathways: insulin/IGF-1, FGF23 and Wnt. Future studies will determine the biological effects of these “rejuvenation” factors on SSC biology and as an approach to enhance bone formation and treat osteoporotic bone fragility.

## Clinical approaches for treating skeletal fragility using SSC

**Fig. 1:**
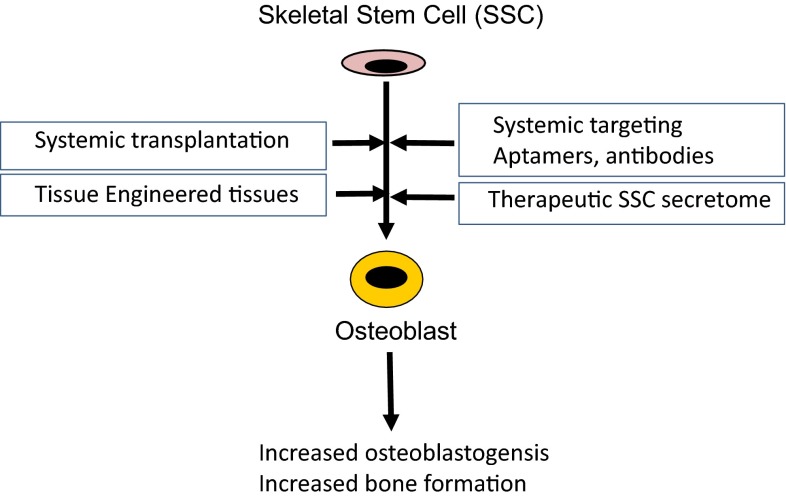
Targeting skeletal stem cells (SSC) for bone formation

 Organ transplantation has been employed with success in modern medicine for treatment of final stages of age-related degenerative diseases e.g. kidney, heart, liver, lung transplant for failing respective organ. However, one disadvantage of this approach is the necessity for using immune suppressive therapy with its accompanying serious side effects of severe infections and risk of cancer development. The use of stem cells in treatment of age-related degenerative diseases has been suggested as an alternative to organ transplantation and with the advantage of possible avoidance of immune suppressive therapy (Kassem 2005). The following are a number of methods where SSC can be targeted to enhance bone formation in vivo (Fig. 1).

### Transplantation of SSC

SSC-based therapeutics has been employed for tissue regeneration and repair of both skeletal and non-skeletal tissues. For skeletal tissue regeneration, the concept is that SSC will home to bone and participate in bone regeneration (Shen et al. 2011). For non-skeletal tissues, the SSC are used as a vehicle for “humoral therapy” delivering their “secretome” consisting of factors that enhance tissue regeneration and repair to injured tissues [please see review in (Caplan and Correa 2011)]. We have recently reviewed the clinical experience with SSC transplantation (Aldahmash et al. 2012). Currently, a number of ongoing clinical trials using culture expanded SSC for localized tissue defects e.g. delayed and non-union fractures, osteonecrosis of femoral head and repair of bone defects associated with maxillary cyst removal (please see: https://clinicaltrials.gov). In some of the current trials, SSC are loaded on biomaterial matrices (scaffold). The biomaterial available for bone tissue regeneration can be classified as either biologically-derived polymers isolated from extracellular matrix, plants or seaweeds e.g. collagen, fibronectin, alginate or synthetic material e.g. hydroxyapatite, tricalcium phosphate ceramics, polylactide and polyglycolide or a combination of these. It is also possible to “functionalize” the scaffold adding a biological material e.g. siRNA, miRNA or a small molecule that can direct the differentiation of stem cells or SSCs into bone lineage (Andersen et al. 2010). While non-healing fractures can be caused by aging and osteoporosis, the use of SSC to enhance bone formation in a systemic bone disease like osteoporosis has not been tried and may not be feasible at present (Aldahmash et al. 2012). Interestingly a report regarding the successful treatment of a genetic form of osteoporosis: osteogensis imperfecata, with intravenous infusion of SSC, has been published (Horwitz et al. 2002). However, this study needs further confirmation in a larger number of patients.

### Targeting SSC in vivo

As an alternative approach for transplantation of in vitro expanded and differentiated SSC, is to target the resident SSC. This approach is clinically relevant for treatment of age-related impaired bone formation. As mentioned above, in the elderly and osteoporotic patients, SSC can respond to biological stimuli. In order to target SSC specifically, a large number of studies have tried to identify molecules that are enriched in SSC populations using global proteome analysis (Kristensen et al. 2012), global gene expression analysis (Twine et al. 2014) or global miRNA gene expression profiling (Eskildsen et al. 2011) that have provided a number of possible targets with effects on proliferation and differentiation of SSC. However the challenge is how to deliver these molecules to SSC in vivo. The following ideas have been tested and reported. Guan et al developed a method to direct SSC to the bone surfaces by linking a synthetic peptidomimetic ligand (LLP2A) directed against integrin a4b1 epitope present on SSC plasma membrane, to a bisphosphonate that has a high affinity for bone (Guan et al. 2012). In mice models, the authors demonstrated the ability of SSC to home to bone and to exert significant enhancement of bone formation (Guan et al. 2012). Interestingly, intravenous injection of the coupled ligand (LLP2A and bisphosphonate) alone in ovariectomized mice (a model of osteoporotic bone loss) increased osteoblast numbers and bone formation, providing a proof-of-concept for ability to target endogenous SSC. Also, two other research groups developed osteoblast and SSC specific aptamers and tested their efficiency in targeting siRNA and miRNA to bone cells. Liang et al developed an osteoblast specific aptamer (CH6) and developed CH6 aptamer-functionalized lipid nanoparticles (LNPs) encapsulating a siRNA targeting pleckstrin homology domain-containing family O member 1 (Plekho1) known to enhance osteoblast function (Liang et al. 2015). The authors reported increased bone formation and bone mass following systemic delivery. Li et al (Li et al. 2015) demonstrated the possibility of using a SSC-specific aptamer delivery system coupled to an inhibitor of miR-188 (aptamer-antagomiR-188) and injected in the intramedullary cavity of mice bone, led to delivery of antagomiR-188 to endogenous SSC and increased bone formation. A similar approach can be used to target small molecules with SSC-specific enhancing effects on differentiation into osteoblasts, can be employed (Jafari et al. 2015). However, no studies have been conducted using these approaches in aged animals.

### Final remarks

The contribution of senescent SSC to skeletal aging is increasingly recognized, and supported by a large number of in vitro and in vivo studies. Recent studies suggest that extrinsic factors present in the aging microenvironment play a dominant role in impairing SSC functions during aging. These studies also show that SSC obtained from elderly persons and patients with osteoporosis maintain responses to extrinsic stimuli. Thus, “rejuvenation” of SSC is possible treatment option for age-related skeletal diseases. Advances in identification of rejuvenating molecules, molecular targets and in vivo systemic delivery systems targeting SSC, will enable the use of these novel therapies in clinical practice.
